# Supplementary material for: Correlation between baseline anion gap and early acute kidney injury in patients with acute pancreatitis in the intensive care unit: A single-center retrospective cohort study
Source: PLoS One. 2025 Feb 11;20(2):e0315386. doi: 10.1371/journal.pone.0315386 (PMC11813115; doi:10.1371/journal.pone.0315386)
Supplement: S1 Table — (DOCX) [file pone.0315386.s001.docx]

**S1 Table. Relationship between threshold inflection point and AKI.**

| **Variable** | **OR (95%CI)** | ***P*** |
| --- | --- | --- |
| AG<15mmol/L | 0.809 (0.595~1.098) | 0.1738 |
| 15mmol/L≤AG<28mmol/L | 1.253 (1.136~1.383) | <0.001 |
| AG≥28mmol/L | 0.868 (0.741~1.018) | 0.0822 |

Inflection point1:15mmol/L;

Inflection point2:28mmol/L.
