# Supplementary material for: Correlation between baseline anion gap and early acute kidney injury in patients with acute pancreatitis in the intensive care unit: A single-center retrospective cohort study
Source: PLoS One. 2025 Feb 11;20(2):e0315386. doi: 10.1371/journal.pone.0315386 (PMC11813115; doi:10.1371/journal.pone.0315386)
Supplement: Attachment — Submitted filename: Response to Reviewers.docx [file pone.0315386.s004.docx]

Dear Editor Keiko Hosohata and Reviewers，

On behalf of my co-authors, I would like to extend our sincerest gratitude to you and the reviewers for the meticulous attention and valuable feedback provided during the review process of our manuscript titled “Relationship between serum anion gap and acute kidney injury in patients with acute pancreatitis upon admission: a retrospective study based on MIMIC-IV database” (PONE-D-23-43806).

The thoughtful comments and suggestions have greatly enhanced the robustness and clarity of our work. In compliance with the reviewers’ recommendations, we have individually addressed each comment and revised the manuscript accordingly. All authors have thoroughly reviewed the revised version and given their approval for submission to PLOS ONE.

We look forward to your positive response to this submitted revision. If we can be of any further assistance, please do not hesitate to contact us.

Thank you once again for your support and guidance throughout this process.

**Responds to the reviewer’s comments:**

**Reply to Reviewer #3**

Dear Reviewer,

We sincerely thank you for your time and effort in reviewing our manuscript. Your keen comments and suggestions have been invaluable to our research, and we are pleased to learn that our modifications have been approved by you.

We greatly appreciate your support throughout this process and are cautiously optimistic that the improvements we have implemented will yield good results for our manuscript.

With anticipation, we look forward to the next stage of our publishing journey and are hopeful of a positive outcome that reflects the hard work and dedication we have put into this project.

Thank you again for your insightful contribution to our work.

**Reply to Reviewer #4**

Dear Reviewer,

First and foremost, we would like to extend our gratitude for your thorough review and the positive feedback you have provided. We are committed to improving the quality of our manuscript, and we have taken your suggestions into careful consideration. Below, we address the specific comments you have made:

**Response to Comment 1:**

We are glad to hear that the grammar of our manuscript has improved. We have conducted a further review and made additional grammatical corrections throughout the text, including in the flow diagram. We have also ensured that the language flows smoothly and coherently to enhance readability. We believe these additional corrections will meet your expectations and we look forward to your assessment of the revised flow diagram.

**Response to Comment 2:**

We appreciate your suggestion regarding the flow diagram. As per your advice, we have removed the reference to "excluded participants age <18 years n=0" and have instead included a clear statement in the methods section. This now explicitly states that our study population consists solely of individuals aged 18 years or older, thereby streamlining the flow diagram and enhancing the clarity of our methods.Here is the latest flow chart(Fig1).

**Fig1.** Flowchart exhibiting the selection process.

We have uploaded all image files to the Preflight Analysis and Conversion Engine (PACE) digital diagnostic tool.

We hope that these revisions are in line with your suggestions and that they will lead to a more polished and precise manuscript. We eagerly await your feedback and are hopeful for a positive outcome.

Thank you once again for your insightful comments and for helping us to improve our work.

Warm regards,

Jinfeng Li

E-mail:wcwkljf@163.com

Affiliated Hospital of Jining Medical University, NO.89,Guhuai Road,Rencheng District,Jining, Shandong, China
